# Gallbladder volvulus diagnosed by MRCP and diffusion-weighted imaging in an elderly woman

**DOI:** 10.1016/j.radcr.2025.11.051

**Published:** 2025-12-17

**Authors:** Isaac Azad, Ivan Arsic, Iben Gad Lauridsen, Tobias Stadil

**Affiliations:** a Department of Radiology, Aalborg Hospital, Hobrovej 18-22, 9000 Aalborg, Denmark; b Department of Radiology, Viborg Hospital, Heibergs Allé 4, 8800 Viborg, Denmark; c Department of General surgery, Viborg Hospital, Heibergs Alle 5A, 8800 Viborg, Denmark

**Keywords:** Gallbladder (GB) volvulus, MRCP, DWI, Whirl sign, Ischemic cholecystitis

## Abstract

Gallbladder (GB) volvulus is a rare cause of acute abdominal pain and is frequently misdiagnosed as acute cholecystitis due to its nonspecific presentation. We report an elderly woman in whom magnetic resonance cholangiopancreatography (MRCP) with diffusion-weighted imaging (DWI) enabled a timely, accurate preoperative diagnosis. An 84-year-old woman presented with acute right upper quadrant pain; inflammatory markers were normal. Ultrasound (US) and contrast-enhanced computed tomography (CT) suggested acalculous cholecystitis; CT showed a “whirl sign” but no V-shaped distortion of the extrahepatic ducts. MRCP confirmed a “whirl sign” involving the cystic duct and artery, and DWI demonstrated diffusion restriction in the GB wall, consistent with acute ischemia secondary to torsion. Laparoscopic cholecystectomy confirmed a 360° torsion with ischemic changes. This case highlights the limitations of US and the variable sensitivity of CT for GB volvulus, especially in atypical presentations. MRCP with DWI provides complementary anatomic and functional information, supporting a confident preoperative diagnosis and prompt surgical intervention.

## Introduction

Gallbladder (GB) volvulus is an exceedingly rare condition, with a reported incidence of approximately 1 in 365,520 hospital admissions. Since its first description in 1898, fewer than 600 cases have been documented [[1], [2], [3]]. It predominantly affects elderly women and typically presents with symptoms such as right upper quadrant pain, nausea, and vomiting, mimicking acute cholecystitis; this clinical overlap contributes to a low rate of correct preoperative diagnosis, historically between 10% and 26% [1,2].

The diagnostic challenge is often compounded by atypical presentations. Notably, up to 45% of patients may present without elevated inflammatory markers, making it difficult to differentiate GB volvulus from other acute abdominal conditions [2]. Anatomic predispositions for GB volvulus include an elongated mesentery, which increases GB mobility, and age-related liver atrophy or loss of visceral fat, which can result in a “floating GB” [2].

Ultrasound (US) and computed tomography (CT) are the standard first-line imaging modalities, but their findings in GB volvulus, such as GB distension, wall thickening, and pericholecystic fluid, are often nonspecific. Magnetic resonance cholangiopancreatography (MRCP) provides superior sensitivity for biliary pathology, with recent studies reporting 95&-100% sensitivity for gallstone detection [4]. When combined with diffusion-weighted imaging (DWI), which is highly sensitive for detecting acute ischemic changes [5], this advanced imaging approach can be pivotal for an accurate diagnosis. We present a case illustrating the diagnostic value of MRCP with DWI in achieving a precise preoperative diagnosis of GB volvulus in a patient with a misleading clinical picture and, to our knowledge, this is among the first reports in the literature utilizing combined MRCP and DWI to diagnose GB volvulus complicated by acute ischemic cholecystitis.

## Case presentation

An 84-year-old woman presented with a 1-month history of intermittent upper abdominal pain that acutely worsened over the preceding 24 hours. Past medical history included perforated diverticulitis managed in 2021. She was afebrile (37.3°C) with right upper quadrant tenderness and guarding. She denied nausea, vomiting, and altered bowel habits.

Laboratory tests showed a normal white blood cell count and C-reactive protein, with mildly elevated total bilirubin (28 µmol/L), lactate dehydrogenase (385 U/L), and alanine aminotransferase (229 U/L). Alkaline phosphatase was normal (92 U/L).

**Fig. 1:**
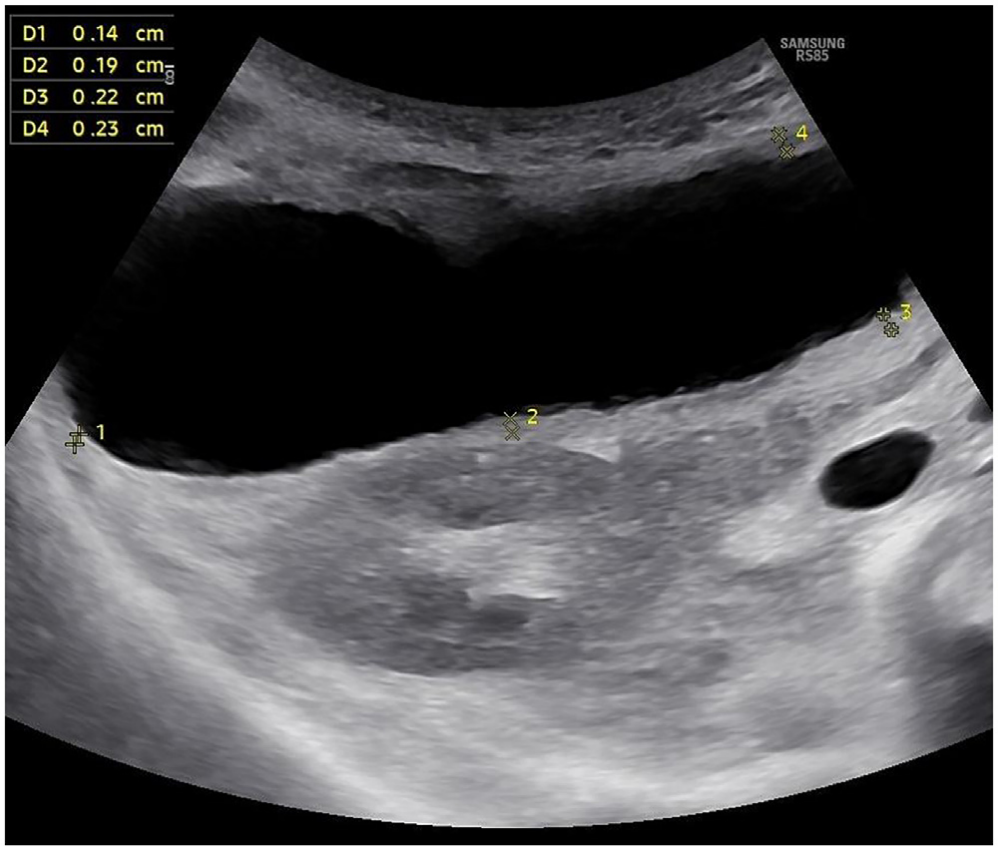
Ultrasound showing a distended gallbladder with normal wall thickness and no gallstones. Wall thickness is indicated in mm with yellow annotations.

**Fig. 2:**
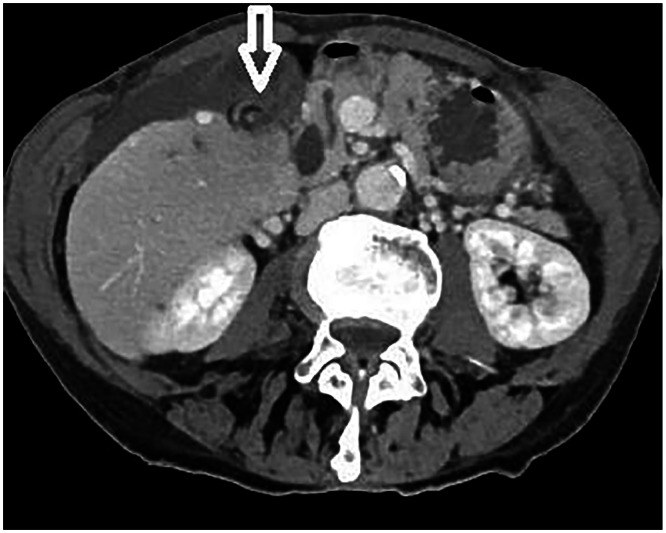
Portal venous phase CT showing the whirl sign (white arrow) indicative of gallbladder torsion.

Abdominal US demonstrated a distended GB with wall thickness of 3 mm and a small amount of pericholecystic fluid; no gallstones were visualized (Fig. 1). Portal-venous-phase contrast-enhanced CT confirmed GB distension and diffuse wall enhancement, with a dilated common bile duct (CBD) measuring 11 mm. A “whirl sign” suggested torsion, although there was no V shaped distortion of the extrahepatic ducts (Fig. 2). No intraductal stones, strictures, or masses were identified.

**Fig. 3:**
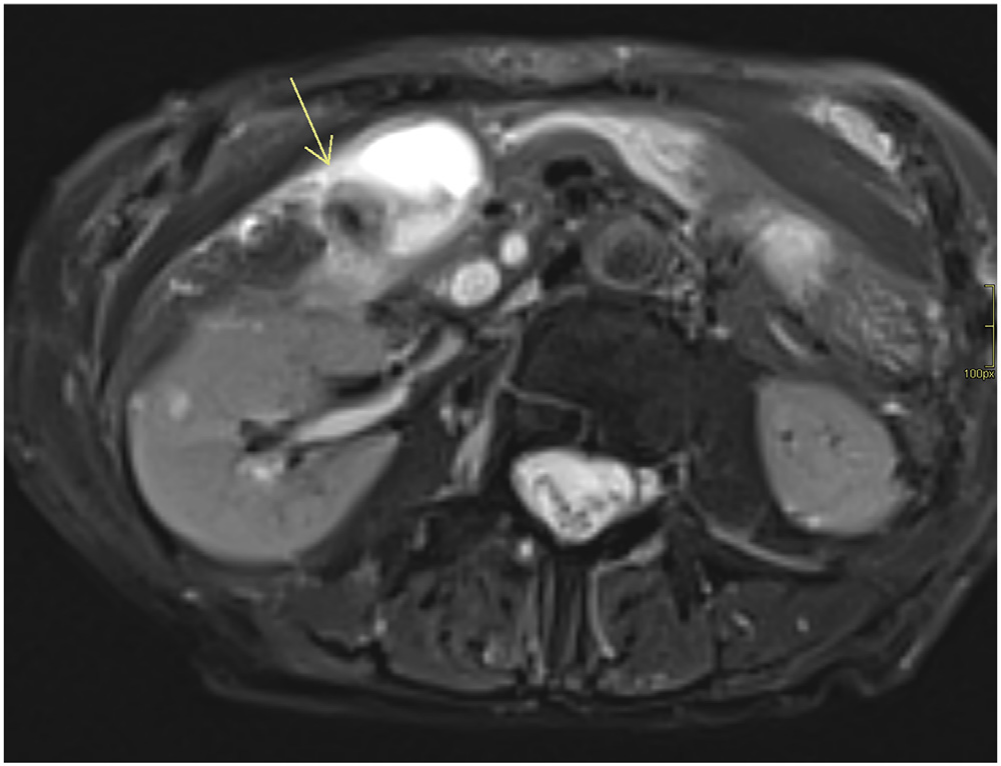
Axial T2-weighted MRCP image showing a distended gallbladder with significant wall edema and a “whirl sign” (arrow) involving the cystic duct and artery, indicative of torsion.

**Fig. 4:**
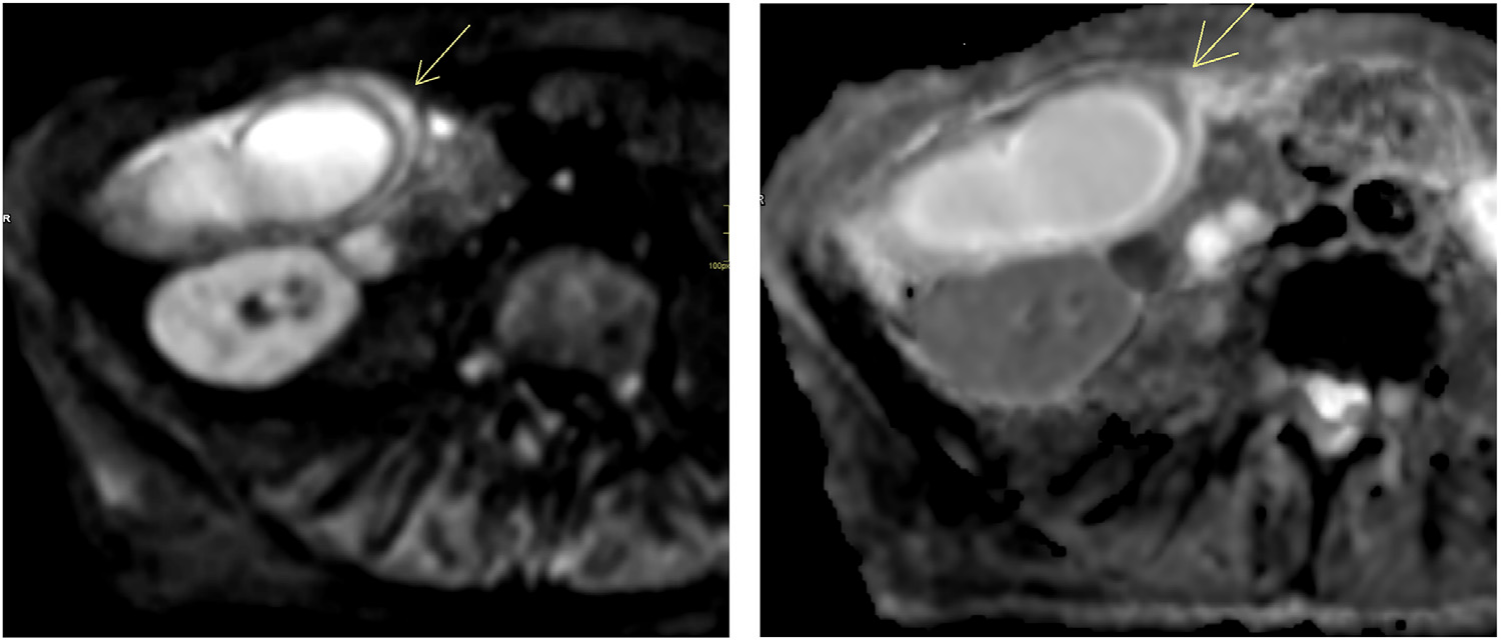
(A) Diffusion-weighted imaging (DWI) sequence (b800) demonstrating pronounced hyperintensity in the gallbladder wall (arrow). (B) Corresponding apparent diffusion coefficient (ADC) map showing diffuse hypointensity, consistent with true diffusion restriction and acute ischemia.

MRCP with DWI was performed the same day as US and CT to further assess the biliary system, specifically to clarify the cause of CBD dilatation and suspected torsion. MRCP demonstrated significant GB wall edema, pericholecystic free fluid, and a characteristic “whirl sign” involving the cystic duct and artery, which is a specific indicator of torsion (Fig. 3). On DWI (b = 800 s/mm²), the GB wall was hyperintense with corresponding low signal on the apparent diffusion coefficient (ADC) map, indicating true diffusion restriction consistent with acute ischemia (Fig. 4). No masses, strictures, polyps, or gallstones were seen.

**Fig. 5:**
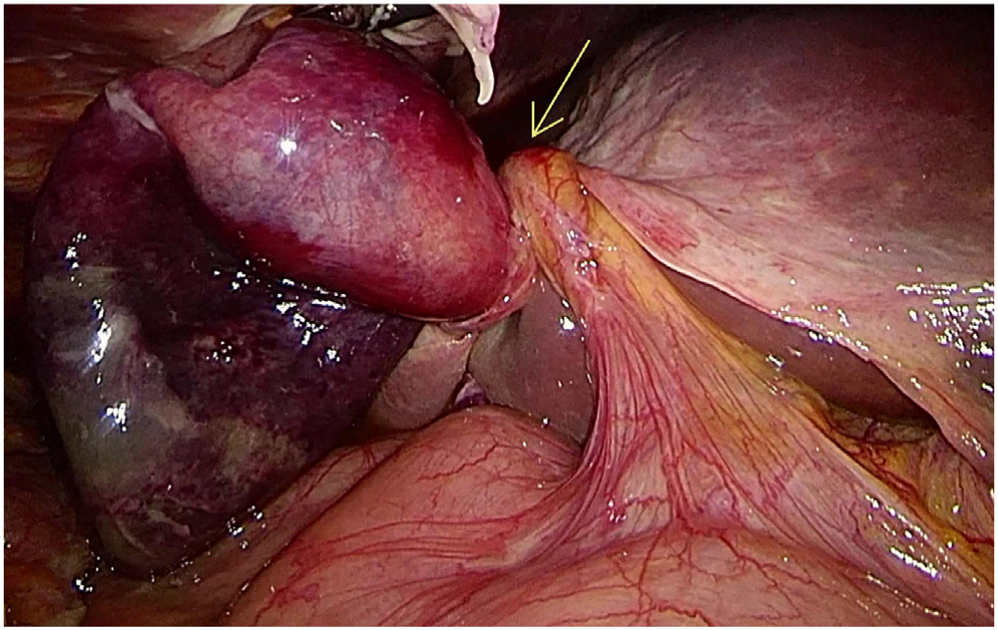
Intraoperative laparoscopic photograph revealing a distended gallbladder detached from the liver bed, with a 360-degree torsion around its pedicle (arrow).

Based on the definitive MRCP and DWI findings, a preoperative diagnosis of GB volvulus with secondary acute ischemia was made. The patient underwent an urgent laparoscopic cholecystectomy, 2 hours and 45 minutes after MRCP. Intraoperative laparoscopic findings confirmed a GB detached from the liver bed with a 360° torsion of its pedicle (Fig. 5). Histopathological examination confirmed acute ischemic necrosis without gallstones or malignancy, consistent with the imaging and surgical findings.

## Discussion

GB volvulus remains a challenging diagnosis due to its rarity and resemblance to acute cholecystitis. In this case, US revealed a GB with normal wall thickness and no gallstones—findings that, together with normal inflammatory markers and afebrile status, typically argue against acute cholecystitis. However, the presence of GB distension and a positive sonographic Murphy’s sign raised suspicion of acalculous cholecystitis [6]. The underlying torsion of the GB was not detected on US.

Portal-venous-phase CT performed under our emergency protocol detected the “whirl sign” (Fig. 2), indicating torsion of the cystic duct and mesentery, a finding reported as a key diagnostic indicator in some reports [7,8]. However, other supportive CT findings, such as V-shaped distortion of the extrahepatic ducts and GB wall edema were absent, underscoring CT’s variable sensitivity [9]. Arterial-phase CT can improve visualization of GB wall edema and help suggest ischemic cholecystitis secondary to torsion [10,11]; however, the etiology of CBD dilatation often remains unclear. This transient, nonobstructive CBD dilatation may share a mechanism with postcholecystectomy biliary changes, as previously described [12], although it remains uninvestigated in GB volvulus.

Given the clinical–radiologic mismatch, MRCP with DWI was instrumental. MRCP delineated the “whirl sign” of the cystic duct and artery, enabling a confident preoperative diagnosis, consistent with previous reports of MRCP’s utility in GB torsion [13]. Importantly, the addition of DWI provided functional information by revealing diffusion restriction in the GB wall, consistent with acute ischemia—the most critical complication of torsion. This finding corroborates other reports on the utility of DWI for diagnosing biliary torsion [14].

While a “whirl sign” on MRCP may suffice in some cases, the addition of DWI enhances diagnostic confidence, helps exclude alternative pathologies such as malignancy, and supports timely surgical management. Although DWI marginally extends scan time (typically 3-5 minutes), its ability to provide an earlier and more definitive diagnosis is crucial, as delayed recognition of GB volvulus can lead to ischemia, necrosis, perforation, peritonitis, and mortality of 5%-10% in untreated cases [15,16].

This patient’s afebrile status and normal inflammatory markers exemplify an atypical yet not uncommon presentation [1,2]. The absence of a typical inflammatory response underscores the need for a high index of suspicion and the judicious use of advanced imaging when initial findings are inconclusive. Timely recognition via advanced imaging like MRCP with DWI can support early laparoscopic cholecystectomy, which is associated with lower complication rates compared with delayed intervention (20–30%) [17]. MRCP with DWI effectively overcomes the diagnostic limitations of US and CT by providing both the anatomic and functional insights necessary for a timely, accurate diagnosis and appropriate surgical management.

## Conclusion

This case highlights the diagnostic value of MRCP combined with DWI in diagnosing GB volvulus, particularly when US is inconclusive and CT findings are incomplete. Clear visualization of the “whirl sign” on MRCP and confirmation of acute ischemia via diffusion restriction on DWI enabled a confident preoperative diagnosis, which supported timely surgical management in an elderly patient. In cases presenting with atypical features of acute cholecystitis, MRCP with DWI should be considered to enhance diagnostic accuracy and contribute to improved patient outcomes.

## Patient consent

To the Publisher: This form should NOT be submitted to *Radiology Case Reports* due to patient confidentiality but is recommended to be stored in the patient’s medical record. By signing this consent statement for publication in *Radiology Case Reports*, the publisher confirms that the necessary consents have been obtained for the publication of photos/illustrations/videos and/or text related to the patient.

## Appendix Supplementary materials

Image, application 1

## References

[1] Reilly D.J., Kalogeropoulos G., Thiruchelvam D. (2012). Torsion of the gallbladder: a systematic review. HPB (Oxford).

[2] Kashyap S., Mathew G., Abdul W., Patel P. (2025). StatPearls.

[3] Edokpolo B., Shaw JW. (2025). Gallbladder volvulus mimicking an intra-abdominal malignancy in an elderly patient on warfarin: a case report. Cureus.

[4] Evanson D.J., Elcic L., Uyeda J.W., Zulfiqar M. (2025). Imaging of gallstones and complications. Curr Probl Diagn Radiol.

[5] Tomizawa M., Shinozaki F., Tanaka S., Motoyoshi Y., Sugiyama T., Yamamoto S. (2017). Diffusion-weighted whole-body magnetic resonance imaging with background body signal suppression/T2 image fusion for the diagnosis of acute cholecystitis. Exp Ther Med.

[6] Yokoe M., Hata J., Takada T., Strasberg S.M., Asbun H.J., Wakabayashi G. (2018). Tokyo Guidelines 2018: diagnostic criteria and severity grading of acute cholecystitis (with videos). J Hepatobiliary Pancreat Sci.

[7] Sato O., Kotani T., Kanayama T., Tokuda B., Yamada K. (2023). Utility of hyperdense whirl sign for the diagnosis of gallbladder torsion. Acta Radiol Open.

[8] Tajima Y., Tsuneoka N., Kuroki T., Kanematsu T. (2009). Clinical images. Gallbladder torsion showing a “whirl sign” on a multidetector computed tomography scan. Am J Surg.

[9] Moriwaki Y., Otani J., Okuda J., Niwano T. (2019). Gallbladder torsion: US, CT, and MRI findings. J Gastrointest Surg.

[10] Uemura S., Higuchi R., Yazawa T., Ota T., Oishi T., Tabata M. (2019). Impact of transient hepatic attenuation differences on computed tomography scans in the diagnosis of acute gangrenous cholecystitis. J Hepatobiliary Pancreat Sci.

[11] Pham HD, Pham HV, Huynh QH. A gallbladder volvulus presenting as acute cholecystitis in a young woman. Cureus. 2020;12(7):e9435. doi:10.7759/cureus.9435. 10.7759/cureus.9435 PMC7450884 32864259

[12] Ahn S.H., An C., Kim S.S., Park S. (2024). CT evaluation of longterm changes in common bile duct diameter after cholecystectomy. J Korean Soc Radiol.

[13] Usui M., Matsuda S., Suzuki H., Ogura Y. (2000). Preoperative diagnosis of gallbladder torsion by magnetic resonance cholangiopancreatography. Scand J Gastroenterol.

[14] Moriwaki Y., Otani J., Okuda J., Niwano T. (2019). Utility of diffusion-weighted imaging for the diagnosis of gallbladder torsion. World J Surg.

[15] David R., Traeger L., McDonald C. (2019). Gall bladder torsion: a disease of the elderly. BMJ Case Rep.

[16] Liu H.T., Yu C.C., Wu C.C., Hwang JI. (2013). Gallbladder volvulus treated by laparoscopic cholecystectomy. Formos J Surg.

[17] Vo N.T., Le V.T., Nguyen QV. (2024). Gallbladder volvulus misdiagnosed as acute acalculous cholecystitis: a case report. Int J Surg Case Rep.

