# Supplementary material for: Gallbladder volvulus diagnosed by MRCP and diffusion-weighted imaging in an elderly woman
Source: Radiol Case Rep. 2025 Dec 17;21(3):1127–30. doi: 10.1016/j.radcr.2025.11.051 (PMC12770950; doi:10.1016/j.radcr.2025.11.051)
Supplement: Supplementary file 1 [file mmc1.pdf]

Samtykkeerklæring - Ugeskrift for Læger

Til forfatter: Denne formular skal IKKE indsendes til Ugeskrift for Læger, af hensyn til patientfortrolighed, men anbefales gemt i patientens journal. I Forfatterskabserklæring vedr. publicering i Ugeskrift for Læger erklærer forfatter(e), at der er indhentet de nødvendige samtykker til publicering af fotos/illustrationer/videoer med og/eller tekst om patienten.

Jeg, [*patientens navn*] Ingrid Pretzmann Jensen

tillader at man i Ugeskrift for Læger publicerer fotos/illustrationer/videoer af mig og/eller tekst, som omhandler mig og som kan være genkendelig for andre. Jeg er indforstået med, at det vil blive bragt uden mit navn og så anonymiseret som muligt, men at der ikke kan garanteres for at sundhedspersonale fra mit behandlingsforløb, eller pårørende, ikke vil kunne genkende min historie.

Fotos/illustrationer/videoer af mig og/eller tekst kan publiceres både på tryk i Ugeskrift for Læger og online på Ugeskriftet.dk, som primært læses af læger, men som også er tilgængeligt for ikke-læger, almindelige borgere og journalister. Jeg er indforstået med, hvad det indebærer. Ugeskrift for Læger vil ikke bringe oplysninger fra artiklen i andre sammenhænge end den videnskabelige publicering.

Dette samtykke kan trækkes tilbage, indtil artiklen er antaget til publicering på Ugeskrift for Læger. Herefter kan samtykke ikke trækkes tilbage.

Fotos/illustrationer/videoer viser: galdeblære og omkringliggende væv.

Artiklen omhandler: galdeblære volvulus / snoet/drejet galdeblære.

Artiklens kontaktforfatter: Iben Gad Lauridsen

Patientens navn: Ingrid Pretzmann Jensen

Patientens fødselsdato: 150440 - 1290

Dato: 7/3-25 Patientens underskrift: Ingrid Pretzmann Jensen

Pårørende/værge (på vegne af patienten):

Navn: ______________ Relation til patienten: ______________

Dato: ______ Pårørende/værges underskrift: ______________
